# O-GlcNAcylation of eNOS in high-salt-induced thoracic aorta endothelial dysfunction in mice

**DOI:** 10.3389/fphar.2025.1730447

**Published:** 2025-12-16

**Authors:** Chang Li, Liuming Gao, Yi Ling, Zhan Lv

**Affiliations:** 1 Department of Cardiology, Zhongnan Hospital of Wuhan University, Wuhan, China; 2 Department of Internal Medicine, Zhongnan Hospital of Wuhan University, Wuhan, China; 3 Department of Cardiology, The First Affiliated Hospital with Nanjing Medical University, Nanjing, China

**Keywords:** diastolic blood pressure, endothelial dysfunction, eNOS, high salt diet, O-GlcNAc

## Abstract

**Background:**

Excessive salt intake is a well-established risk factor for hypertension. However, the molecular mechanisms by which salt-induced endothelial dysfunction contributes to blood pressure elevation remain incompletely understood.

**Methods and Results:**

In this study, mice were fed a normal-salt diet (NSD) or high-salt diet (HSD) for 4 weeks, and primary bovine aortic endothelial cells (BAECs) were treated with varying concentrations of NaCl. HSD mice showed increased diastolic blood pressure and impaired acetylcholine-induced vasodilation, while sodium nitroprusside responses remained intact. *In vitro* experiments further confirmed salt-induced vascular endothelial dysfunction, high NaCl treatment reduced the proliferation, migration, and tube formation abilities of BAECs. Western blot analysis revealed that high salt exposure significantly increased O-GlcNAc modification of eNOS and upregulated O-GlcNAc transferase (OGT) expression, without altering total eNOS protein levels. Notably, nitric oxide (NO) bioavailability was significantly reduced both *in vivo* and *in vitro*. Treatment with the OGT inhibitor OSMI-1 reversed these changes and restored endothelium-dependent relaxation in HSD mice.

**Conclusion:**

Our findings suggest that high salt intake impairs endothelial function by enhancing O-GlcNAc of eNOS, thereby contributing to elevated diastolic blood pressure. The ability of OGT inhibition to reverse endothelial dysfunction highlights the therapeutic potential of targeting eNOS O-GlcNAc could be a promising approach for preventing salt-induced vascular damage and subsequent diastolic blood pressure elevation.

## Introduction

Epidemiological and experimental data have provided compelling evidence that high salt is an important factor in the development and progression of cardiovascular disease (Zhang et al., 2025; Yakoub et al., 2024; Powles et al., 2013; Collaborators, 2019). Numerous studies have shown that high-salt diets induce endothelial dysfunction, increase vascular resistance, and elevate blood pressure (Zhou et al., 2020; Wang et al., 2025; He FJ. et al., 2020; He and Macgregor, 2010). The vascular endothelium, a critical regulator of vascular tone and structure, is particularly sensitive to environmental stimuli, including dietary factors such as sodium load.

Endothelial nitric oxide synthase (eNOS) plays a crucial role in preserving vascular integrity through the production of nitric oxide (NO), a potent vasodilator and anti-inflammatory molecule (Liu et al., 2025). The activity of eNOS is tightly controlled by post-translational modifications, among which O-GlcNAc—the addition of O-linked N-acetylglucosamine groups to serine or threonine amino acids (O-GlcNAc)—has recently emerged as a crucial regulatory mechanism (Troiano et al., 2021; He A. et al., 2020). This dynamic alteration is facilitated by the enzyme O-GlcNAc transferase (OGT) and is counteracted by O-GlcNAcase (OGA), has been shown to inhibit eNOS activity by interfering with its phosphorylation and cofactor interactions (Ji et al., 2025). However, the role of eNOS O-GlcNAc in salt-induced vascular dysfunction remains incompletely understood.

In this study, we observed that excessive salt intake impairs endothelial function by enhancing O-GlcNAc of eNOS, both *in vivo* and *in vitro*. High-salt diet in mice and NaCl treatment in cultured endothelial cells significantly reduced vascular relaxation, proliferation, migration, and angiogenic potential of endothelial cells. Further investigation revealed that excessive NaCl increased eNOS O-GlcNAc and upregulated the expression of OGT. This modification resulted in a reduction of NO release in both serum and cell culture supernatants, thereby contributing to vascular endothelial dysfunction. Notably, pharmacological inhibition of OGT by OSMI-1 (the OGT inhibitor) restored NO production and endothelial function. These findings provide new insight into the molecular basis of salt-related vascular injury and highlight eNOS O-GlcNAc may as a potential therapeutic target.

## Methods

### Animal experiments

All procedures involving mice were conducted in accordance with the approval granted by the Animal Care and Ethical Committee of the Medical Research Institute at Wuhan University. This approval ensures that the research adheres to the guidelines set forth by the National Institutes of Health regarding the ethical treatment and use of laboratory animals. In the course of the experiments, approximately 20 g of male C57BL/6 mice, aged between 6 and 8 weeks, were utilized. Mice of the same age were randomly assigned to experimental groups: normal-salt diet (NSD), high-sodium diet (HSD; 4% NaCl in food + 1% NaCl in drinking water); normal-salt diet in combination with the OGT-antagonist OSMI-1 (NSD + OSMI-1 20 mg/kg/d) and HSD in combination with the OGT-antagonist OSMI-1 (4% NaCl in food + 1% NaCl in drinking water + OSMI-1, 20 mg/kg/d). Control animals assigned to the NSD and HSD groups received intraperitoneal injections of the corresponding vehicle at the same volume and schedule as the OSMI-1–treated groups. The mice were maintained under controlled environmental conditions, specifically subjected to a 12-h light and 12-h dark photoperiod, and were given unrestricted access to food and water throughout the study period.

### OSMI-1 administration

OSMI-1 (Cat. #HY-119738, MedChemExpress, China) was administered intraperitoneally at a dose of 20 mg/kg/day throughout the high-salt diet intervention period. The compound was dissolved in a vehicle solution consisting of DMSO and sterile saline, and the final injection volume was adjusted to 10 mL/kg. Mice in the vehicle control group received matched intraperitoneal injections of the vehicle at identical volumes and schedules. All injections were performed once daily at the same time of day to minimize circadian variation.

### Isolation and culture of primary BAECs

Primary bovine aortic endothelial cells (BAECs) were isolated from fresh bovine thoracic aortas obtained from a local abattoir. After removing connective tissue, the aorta was opened longitudinally, rinsed with PBS, and the endothelial layer was gently scraped to collect cells. The harvested cells were centrifuged, resuspended in endothelial growth medium supplemented with 10% FBS and antibiotics, and seeded onto gelatin-coated culture flasks. BAECs were maintained at 37 °C in 5% CO_2_, and cells between passages 2–5 were used for experiments. BAECs were initially cultured in M-199 medium (Invitrogen, United States). This culture medium was augmented with several essential supplements including 10% fetal bovine serum, 50 µM L-arginine, 100 U/mL of penicillin, 100 µg/mL of streptomycin, and L-glutamine to support optimal cell growth and viability. Prior to initiating the experimental procedures, the cells were kept under controlled standard conditions to ensure their stability and health. Following this maintenance phase, the cells were organized into four distinctive treatment groups for the experiments: control group, high-salt group exposed to 20 mM NaCl, group treated solely with OSMI-1 at a concentration of 25 μM, and combined group that received both high salt and OSMI-1 treatments (20 mM NaCl supplemented with 25 µM OSMI-1). BAECs were exposed to NaCl and OSMI-1 for 24 h.

### ENOS purification

The tissues of the thoracic aorta or the bovine aortic endothelial cells (BAECs) were subjected to homogenization using an ice-cold lysis buffer. This buffer was meticulously prepared to contain a variety of components, specifically 50 mM Tris–HCl adjusted to a pH of 7.4, 1% NP-40, 150 mM NaCl, 1 mM ethylenediaminetetraacetic acid (EDTA), in addition to inhibitors that protect against protease and phosphatase activity (Long et al., 2017). Following this initial step, Centrifugation of the resulting lysates was performed at a gravitational force of 12,000 × g for a duration of 15 min, maintained at a temperature of 4 °C. For each immunoprecipitation reaction, 500 μg of total protein was incubated overnight at 4 °C with 2 μg of anti-eNOS antibody (Cat. #32027, Cell Signaling Technology, United States). The antibody–antigen complexes were captured using 30 μL of Protein A/G agarose beads (Cat. #sc-2003, Santa Cruz Biotechnology), followed by an additional 2 h incubation at 4 °C with gentle rotation. The bead-bound complexes were washed four times with lysis buffer containing 0.1% NP-40 to remove nonspecific binding. After washing, the immunoprecipitated proteins were eluted by boiling the beads in SDS sample buffer for 5 min. O-GlcNAc of eNOS was analyzed by Western blot using an anti–O-GlcNAc antibody. The O-GlcNAc signal intensity was normalized to total immunoprecipitated eNOS to quantify the relative O-GlcNAc level.

### Vascular reactivity assay

After 4 weeks of high-salt diet and drinking water, mice were anesthetized with sodium pentobarbital, and the thoracic aorta was carefully isolated. The vessels were immediately placed in cold phosphate-buffered saline (PBS) (Yadav et al., 2015). Vessels were cut into 2–3 mm rings, each ring was mounted in a wire myograph chamber filled with Krebs-Henseleit Solution (KHS) (Itomine et al., 2001), continuously bubbled with 95% O_2_ and 5% CO_2_ at 37 °C. Resting tension was gradually increased to 1.0 g and equilibrated for 60 min, with KHS replaced every 15 min. Endothelial integrity was verified by assessing relaxation responses to acetylcholine (ACh, 10^−6^ M) following pre-constriction with phenylephrine (PE, 10^−6^ M). Cumulative concentration–response curves to ACh (10^−9^–10^−5^ M) and sodium nitroprusside (SNP, 10^−9^–10^−5^ M) were obtained in rings pre-contracted with PE.

### Blood pressure measurement

After the mice were allowed to rest quietly for 10 min, the tail was inserted into the cuff of a non-invasive blood pressure monitoring system (BP-2010, Softron, Japan) and placed in a thermostatically controlled chamber maintained at 37 °C. Once the mice had adapted to the environment, blood pressure measurements were initiated. Each mouse was measured at least three times, and the average of the valid readings was recorded as the systolic and diastolic blood pressure values.

### Morphological analysis

Thoracic aortae were meticulously excised from mice and placed in 4% paraformaldehyde solution at room temperature, where they were allowed to fix for a minimum of 24 h. Following this fixation period, the specimens were subjected to processing for paraffin embedding, after which they were sliced longitudinally into sections measuring 5 μm in thickness. The slices were subsequently deparaffinized using xylene to prepare them for staining. To evaluate the tissue morphology, hematoxylin-eosin staining was performed, and the resulting stained samples were examined under an upright microscope (Leica, Germany).

### Tube formation assay

Matrigel was thawed at 4 °C, and both 24-well plates and 1 mL pipette tips were pre-cooled under the same conditions. Each well received 250 µL of Matrigel, followed by incubation of the plate at 37 °C with 5% CO_2_ for 30 min to facilitate gel polymerization. BAECs were collected through trypsinization and then resuspended in culture medium at a density of 7 × 10^4^ cells/mL, and seeded onto the solidified Matrigel at 3.5 × 10^4^ cells per well. The cells were cultivated at a temperature of 37 °C, with an environment enriched with 5% CO_2_, for a duration of 6 hours. The number of meshes was quantified with ImageJ software (v1.31).

### *In vitro* scratch assay

BAECs were seeded in 6-well culture plates at a density of 5 × 10^5^ cells per well and allowed to reach 90%–100% confluence. A sterile 200 µL pipette tip was utilized to form a linear wound in the cell monolayer. Cells that had detached were eliminated by gentle washing with PBS, after which culture medium was replaced according to group allocation: the control group received M199 medium containing 2% FBS, while the experimental group was treated with M199 medium containing 2% FBS supplemented with 20 mM NaCl. Wound area images were taken at 0, 12, and 24 h with the aid of an inverted phase-contrast microscope (Olympus, Japan). The movement of BAECs into the wound region was assessed by measuring the remaining wound width or area using ImageJ.

### Transwell migration assay

BAECs were serum-starved for 6 h prior to the assay, then resuspended in serum-free M199 medium and plated into the upper chambers at a cell density of 1 × 10^5^ cells per well (200 μL per insert). In the lower chambers, 600 μL of M199 medium containing either 2% FBS (control group) or 2% FBS with an addition of 20 mM NaCl (NaCl group) was added. The setup was then incubated for 12 h at 37 °C. The migrated cells that adhered to the lower surface were subsequently fixed using a 4% paraformaldehyde solution. Following fixation, the cells were dyed with 0.1% crystal violet and examined under an inverted microscope. Cells that migrated were counted from five randomly chosen microscopic fields on each membrane.

### Western blotting

Proteins were extracted from aortic tissue or BAECs using radioimmunoprecipitation assay (RIPA) buffer and quantified through a BCA assay. The samples underwent denaturation by heating in a metal bath at 100 °C for 10 min, after which equal amounts of protein were subjected to SDS–PAGE, followed by transfer to PVDF membranes. The membranes were blocked for 90 min at room temperature in a solution of 5% BSA dissolved in TBS that contained 0.1% Tween-20, and were then incubated overnight at 4 °C with the specified primary antibodies. After washing, membranes were exposed to HRP-conjugated secondary antibodies for 90 min at room temperature. Protein bands were visualized using enhanced chemiluminescence (ECL) reagent, and images were acquired with a Bio-Rad imaging system (Bio-Rad Laboratories, United States). The primary antibodies used for Western blotting were: anti-eNOS (Cat. #32027, Cell Signaling Technology, United States), anti-O-GlcNAc (Cat. #05-1245, Sigma, United States), anti-OGT (Cat. #ab177941, Abcam, England), anti-OGA (Cat. #ab124807, Abcam, England), anti-eNOS (Cat. #32027, Cell Signaling Technology, United States), anti-p-eNOS Ser1177 (Cat. #9571, Cell Signaling Technology, United States), anti-Caveolin-1 (Cat. #16447-1-AP, Proteintech, China) and anti-GAPDH (Cat. #60004-1, Proteintech, China).

### Measurement of NO

BAECs were plated in 12-well plates (2 × 10^5^ cells/well) in DMEM with 10% bovine serum and cultured until 90% confluence. Then cells were treated with NaCl (0, 20, 40, 60, 80 mM) for 15 min. Afterwards, cell media were collected and cell debris was removed by centrifugation at 12,000 rpm for 20 min. NO levels in serum and cell culture supernatants were determined using a commercial detection kit (Cat. #S0021S, Beyotime, China) based on the modified Griess reaction.

### Serum Na^+^ measurement

Serum Na^+^ concentration was determined using a commercial colorimetric assay kit (Na^+^ Content Assay Kit (Cat. #BC2785, Solarbio, China) according to the manufacturer’s instructions. Briefly, the sodium-activated β-galactosidase catalyzes the substrate p-nitrophenyl-β-D-galactopyranoside (PNPG) to generate p-nitrophenol. The rate of increase in absorbance at 405 nm per unit time is directly proportional to the sodium concentration. The assay was performed on a fully automated biochemical analyzer (Cat. #Spectramax i3x, Molecular Devicesmd, United States). The instrument was calibrated daily using the standard solutions provided with the kit, establishing a standard curve for precise quantification. The final sodium ion concentrations in the serum samples were calculated and are expressed in millimoles per liter (mM).

### Statistical analysis

All experimental data are expressed as the mean value accompanied by the standard error of the mean (SEM). To conduct statistical analyses, we utilized GraphPad Prism software, (version X.0, United States). Normality of data distribution was assessed using the Shapiro-Wilk test. When comparing two separate groups, we employed unpaired Student’s t-tests. For assessments involving multiple groups, one-way ANOVA was implemented, followed by Tukey’s *post hoc* test to determine significance among the groups. *P* < 0.05 was established as the threshold for statistical significance in our analyses.

## Results

### High-salt diet elevates blood pressure and impairs vascular function in mice

**FIGURE 1:**
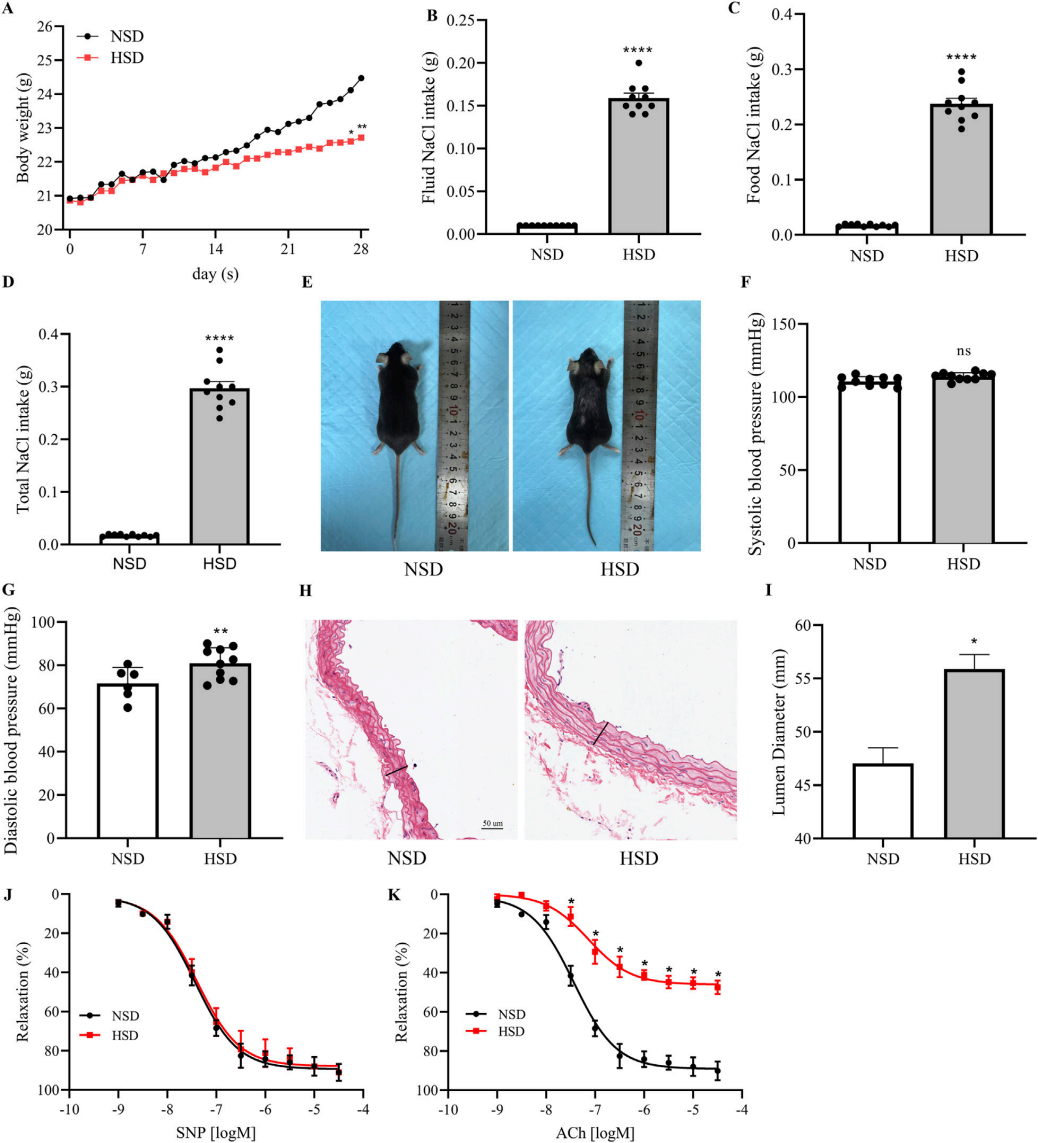
Effects of High-Salt Diet on Blood Pressure and Vascular Function in Mice. **(A)** Body weight changes during the 4-week intervention period in mice fed with a normal-salt diet (NSD) or high-salt diet (HSD). **(B–D)** Quantification of NaCl intake from drinking water **(B)**, food **(C)**, and total NaCl intake **(D)** in NSD and HSD groups. **(E)** Representative images of mice after 4 weeks of high-salt diet intervention. **(F,G)** Systolic **(F)** and diastolic **(G)** blood pressure measurements in mice fed with NSD or HSD for 4 weeks. **(H)** Representative H&E staining of thoracic aorta sections from NSD and HSD mice. Scale bar: 50 µm. **(I)** Quantification of aortic lumen diameter. **(J,K)** Acetylcholine (ACh)-induced relaxation **(J)** and sodium nitroprusside (SNP)-induced relaxation **(K)** of mouse aortas from mice fed with NSD or HSD for 4 weeks. N = 5. *P < 0.05, **P < 0.01, ***P < 0.001, ****P < 0.0001, ns: no significance. Data are mean ± SEM.

Although the detrimental effects of high-salt intake on vascular function are well recognized, the underlying mechanisms remain incompletely understood and are still a matter of debate (Zhou et al., 2020; Wang et al., 2025; He FJ. et al., 2020; He and Macgregor, 2010). To investigate the vascular effects of excessive salt intake, mice were subjected to NSD and HSD for 4 weeks. Starting from day 21, a noticeable difference in body weight emerged between the two groups, with NSD mice gaining significantly more weight than those in the HSD group. By day 28, the difference reached its peak, with a mean weight gap of 1.75 ± 0.46 g (Figure 1A). During the intervention, total NaCl intake, calculated from both chow and drinking water, was significantly higher in the HSD group (Figures 1B–D). In addition, mice in the HSD group exhibited visibly smaller body size and sparser fur compared to those in the NSD group (Figure 1E). Tail-cuff measurements revealed a marked increase in diastolic blood pressure in HSD-fed mice, whereas systolic pressure remained relatively unchanged (Figures 1F,G). Histological analysis of thoracic aortas demonstrated structural alterations in the HSD group, with quantification indicating an increased luminal diameter (Figures 1H,I). To further assess vascular function, *ex vivo* aortic ring assays were performed. The HSD group exhibited a marked decrease in endothelium-dependent relaxation when exposed to acetylcholine (ACh), whereas the response to sodium nitroprusside (SNP), an endothelium-independent vasodilator, was preserved (Figures 1J,K). These findings suggest that high-salt diet induces endothelial dysfunction, primarily through impaired nitric oxide–mediated vasodilation.

### NaCl impairs migration and angiogenic potential of BAECs *in vitro*

**FIGURE 2:**
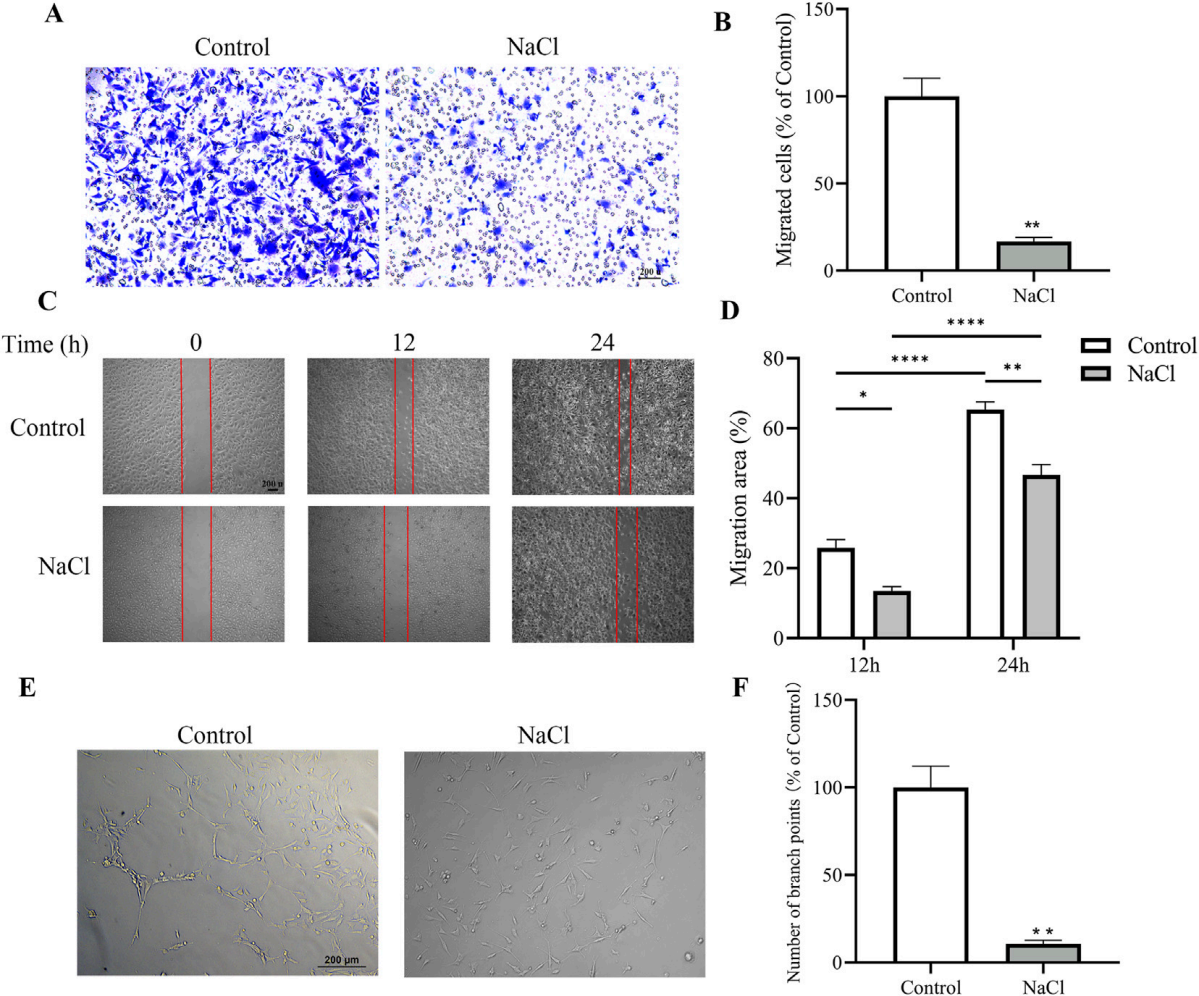
The effects of NaCl treatment on migration and angiogenic capacity of BAECs *in vitro*. **(A,B)** Transwell migration assay showing a significant reduction in the number of migrated BAECs following NaCl treatment compared with control. Representative images **(A)** and quantification **(B)** are shown. **(C,D)** The representative images of scratch assay captured at 0, 12, and 24 h (scale bar = 200 μm) and the migration area (%) calculated according to the formula: migration area (%) = (A0 - At)/A0 × 100%. A0 represented the initial migration area and At represented the remaining migration area when the measurement was done. **(E)** Representative phase-contrast images of capillary-like structures; **(F)** Quantification of the number of branch points formed. N = 5. *P < 0.05, **P < 0.01, ****P < 0.0001. Data are mean ± SEM.

Endothelial cells serve as the main functional element of the vascular wall and are essential for preserving vascular homeostasis (Zuo et al., 2025). To further explore the direct effects of high salt on endothelial cell function, we treated BAECs with NaCl *in vitro* and conducted a series of functional assays. In the transwell migration assay, NaCl-treated cells exhibited a marked reduction in migratory capacity, as evidenced by fewer cells crossing the membrane and reduced crystal violet staining intensity (Figures 2A,B). Wound healing assays showed delayed closure of the scratch area in NaCl-treated cells at 0, 12, and 24 h. Quantitative analysis revealed a significantly lower migration area compared to control cells (Figures 2C,D). In addition, NaCl exposure substantially impaired the ability of BAECs to form capillary-like networks in the tube formation assay, as shown by decreased branch points and disrupted tubular structures under phase-contrast microscopy (Figures 2E,F). Together, these *in vitro* findings demonstrate that elevated extracellular NaCl directly compromises endothelial cell migration and angiogenic capacity, supporting the *in vivo* observation of vascular endothelial dysfunction under high-salt conditions.

### High-salt exposure induces O-GlcNAc of eNOS in vascular tissues and endothelial cells

**FIGURE 3:**
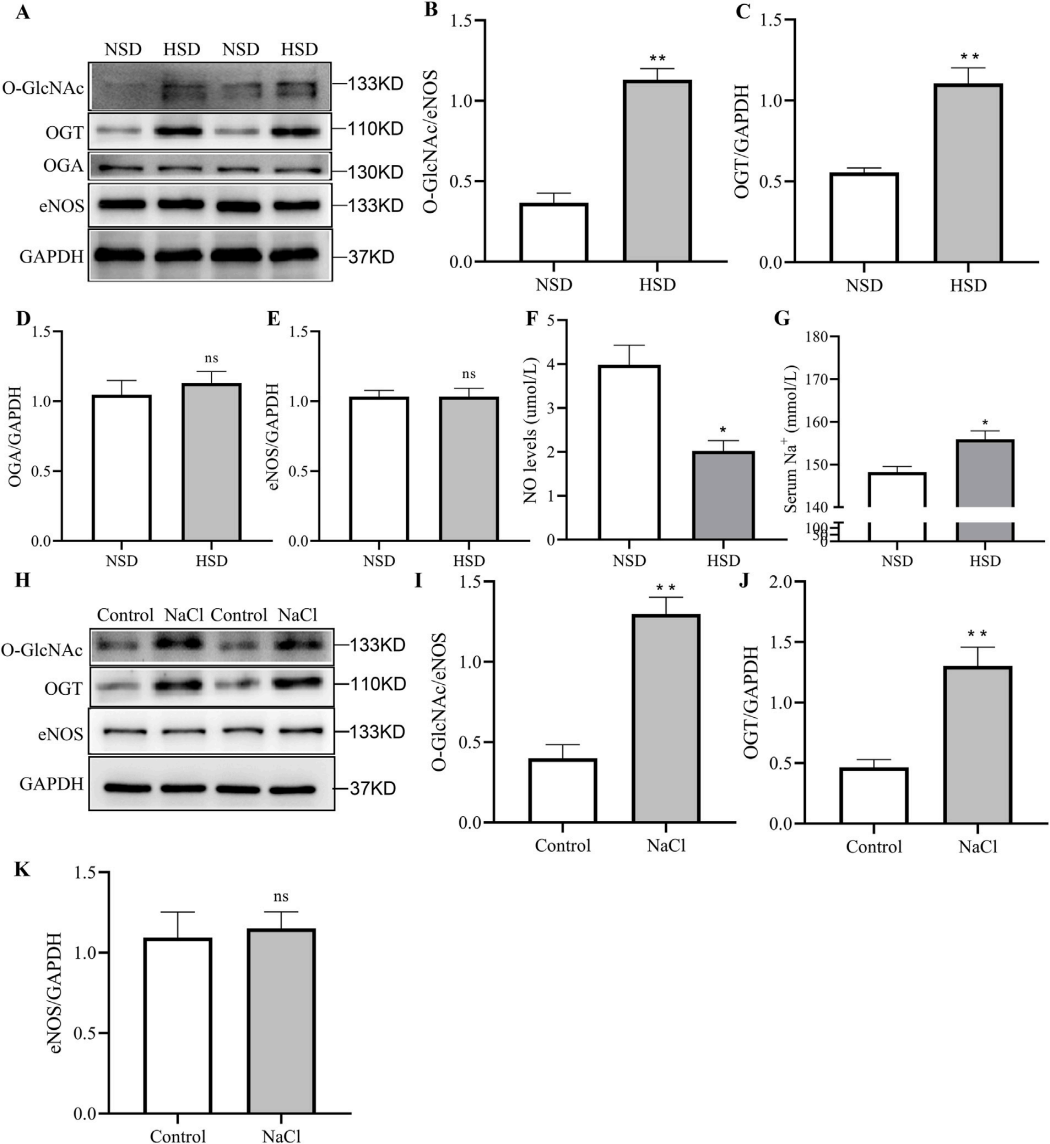
Changes in eNOS O-GlcNAc of mouse aortic tissue and BAECs following high-salt diet or NaCl treatment. Representative Western blot **(A)** images and quantification of O-GlcNAc **(B)**, OGT **(C)**, OGA **(D)** and eNOS **(E)** in mice treated with NSD or HSD for 4 weeks. **(F)** Measurement of serum NO levels in mice fed with NSD and HSD. **(G)** Measurement of serum Na^+^ concentration in mice fed with NSD and HSD. **(H–K)** Representative Western blot **(H)** images and quantification of O-GlcNAc **(I)**, OGT **(J)** and eNOS **(K)** in BAECs treated with NaCl for 24 h. N = 5. *P < 0.05, **P < 0.01, ***P < 0.001, ns: no significance. Data are mean ± SEM.

ENOS is a key enzyme responsible for maintaining vascular homeostasis, primarily through the production of NO, which regulates vasodilation. Recent studies have shown that the activity of eNOS is regulated not only at the transcriptional level but also by various post-translational modifications (PTMs). Environmental stressors such as high salt and high glucose can modulate eNOS activity by altering its glycosylation status. In particular, elevated O-GlcNAc under hyperglycemic conditions has been shown to suppress eNOS activity and reduce NO production (Beleznai and Bagi, 2012). However, whether high salt intake induces similar O-GlcNAc-mediated inhibition of eNOS remains unclear. To further explore the molecular mechanisms underlying high-salt-induced vascular endothelial dysfunction, we assessed the O-GlcNAc modification of eNOS and the expression of related enzymes in mouse aortic tissue and BAECs. Western blot analysis revealed that, compared with the NSD group, mice fed with a HSD exhibited a significant increase in eNOS O-GlcNAc in the aorta (Figures 3A,B), as indicated by the elevated O-GlcNAc/eNOS ratio. In parallel, the expression of OGT was significantly upregulated, while no significant differences were observed in the expression of OGA or total eNOS protein (Figures 3C–E). To further investigate the functional consequences of high salt on eNOS activity, we measured serum NO levels (Figure 3F) in mice. High-salt treatment markedly decreased NO production, supporting the notion that excessive salt intake may promote eNOS O-GlcNAc, inhibit its enzymatic activity, and ultimately impair endothelial function. Compared to the NSD group, high-salt feeding significantly increased Na^+^ concentration in mice by approximately 5% (from 148.2 ± 1.3 mM to 155.9 ± 1.9 mM, *P* < 0.01).

To examine the direct effect of NaCl, BAECs were treated with NaCl *in vitro*. Similar to the *in vivo* findings, NaCl exposure significantly increased the O-GlcNAc/eNOS ratio and OGT expression, without affecting total eNOS levels (Figures 3H–K). Furthermore, NO release into the cell culture supernatant decreased in a dose-dependent manner as NaCl concentrations rose from 0 to 80 mM (Supplementary Figure S3), suggesting that NaCl inhibits eNOS activity, thereby decreasing NO synthesis and contributing to endothelial dysfunction. To control for the potential effects of osmotic pressure, we treated BAECs with mannitol. We found that 80 mM mannitol induced a significant change in NO release, whereas lower concentrations had no effect.

### Inhibition of O-GlcNAc reverses high-salt-induced vascular dysfunction

**FIGURE 4:**
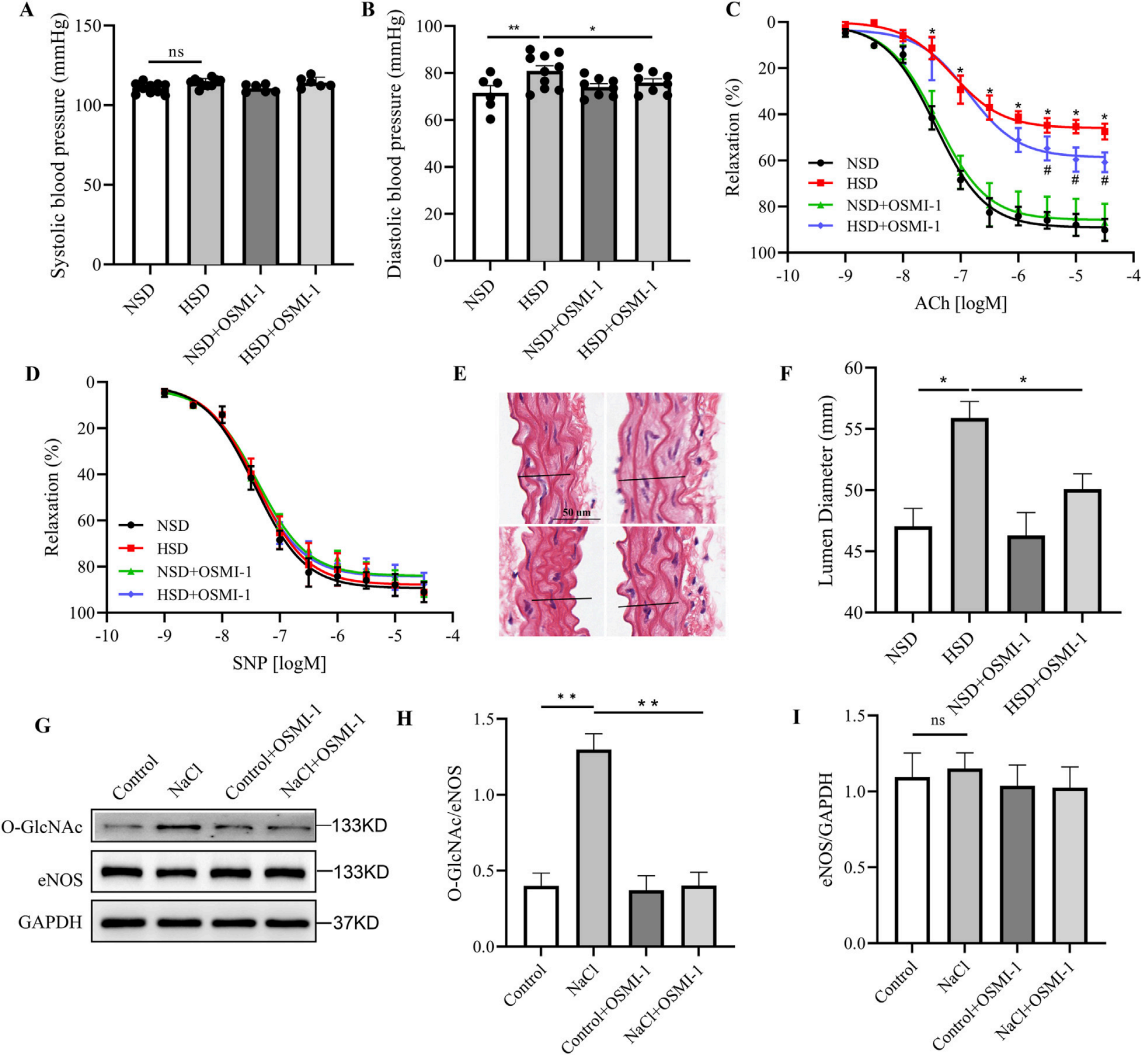
OSMI-1 reversed endothelial injury induced by high-salt diet. **(A,B)** Systolic **(A)** and diastolic **(B)** blood pressure measurements in four groups of mice: NSD, HSD, NSD + OSMI-1, and HSD + OSMI-1. **(C,D)** ACh-induced relaxation **(C)** and SNP-induced relaxation **(D)** of mouse aortas in four groups of mice: NSD, HSD, NSD + OSMI-1, and HSD + OSMI-1. **(E)** Representative H&E staining of thoracic aorta sections in four groups of mice: NSD, HSD, NSD + OSMI-1, and HSD + OSMI-1. Scale bar: 50 µm. **(F)** Quantification of aortic lumen diameter. **(G–I)** Representative Western blot **(G)** images and quantification of O-GlcNAc **(H)** and eNOS **(I)** in four groups of BAECs: Control, NaCl, Control + OSMI-1, and NaCl + OSMI-1. N = 5. *P < 0.05, **P < 0.01, ***P < 0.001, ****P < 0.0001, ns: no significance. Data are mean ± SEM.

To investigate whether O-GlcNAc plays a critical role in salt-induced vascular injury, we administered the selective OGT inhibitor OSMI-1 to mice and evaluated its effect on vascular function. OSMI-1 had no significant effect on systolic blood pressure across groups, but it markedly reduced the elevated diastolic pressure in the HSD group (Figures 4A,B), suggesting a protective role in vascular relaxation regulation. Aortic ring vasodilation experiments further supported this finding. Mice in the HSD group exhibited significantly impaired ACh-induced endothelium-dependent relaxation, whereas co-treatment with OSMI-1 partially restored this response (Figure 4C). In contrast, endothelium-independent vasodilation induced by SNP showed no significant difference among the groups (Figure 4D), indicating that smooth muscle function remained intact and endothelial dysfunction was the primary defect. HE staining revealed an enlarged vascular lumen diameter in the HSD group, which was attenuated by OSMI-1 treatment (Figures 4E,F), further suggesting that O-GlcNAc of eNOS may also contribute to pathological vascular remodeling. Western blot analysis showed that OSMI-1 significantly inhibited NaCl-induced O-GlcNAc of eNOS expression (Figures 4G–I). Collectively, these findings indicate that the abnormal O-GlcNAc modification of eNOS is crucial in the development of endothelial dysfunction caused by high salt levels, and targeting this modification via OGT inhibition may represent a potential therapeutic strategy to alleviate salt-related vascular damage.

## Discussion

Although the association between excessive salt intake and hypertension is well recognized (Alves-Lopes et al., 2025). The precise mechanisms remain only partially clarified. Evidence indicates that consuming a high-salt diet (6,900–8,050 mg sodium/day for 7 days) can attenuate endothelium-dependent vasodilation (Dupont et al., 2013), and prolonged high salt exposure markedly decreases aortic relaxation in response to ACh (Barton et al., 1998).

This study demonstrates that high salt intake impairs vascular endothelial function by promoting aberrant O-GlcNAc of eNOS. Both *in vivo* and *in vitro* experiments revealed that a high-salt diet or NaCl exposure upregulated the expression of OGT, increased eNOS glycosylation levels, and significantly reduced NO bioavailability without altering total eNOS protein expression, ultimately compromising vasodilatory capacity. Importantly, treatment with the OGT inhibitor OSMI-1 effectively reversed these molecular and functional alterations, suggesting that O-GlcNAc may serve as a critical and druggable target in high salt–induced endothelial dysfunction.

Endothelial cells form a selective interface between the bloodstream and underlying tissues, playing key roles in vascular biology, such as regulating inflammation, mediating angiogenesis, and controlling vascular tone through vasoconstriction and vasodilation (Frieden, 2016). Earlier research has shown that excessive dietary salt can reduce NO production and impair endothelium-dependent vasodilation in animal models (Xu et al., 2024; Raffai et al., 2011; Nurkiewicz and Boegehold, 2007). Consistent with these findings, our results demonstrated that after 4 weeks of feeding with a high-salt diet (4% NaCl in chow and 1% NaCl in drinking water), mice exhibited a significant reduction in body weight compared with controls (Figure 1A), elevated diastolic blood pressure (Figure 1G), and marked impairments in endothelium-dependent relaxation responses to ACh (Figure 1H). These findings confirm that high-salt intake induces endothelial dysfunction, primarily by impairing.

The observed reduction specifically in diastolic pressure, without a significant change in systolic pressure, may be attributed to the distinct physiological determinants of each parameter. Diastolic pressure is more strongly influenced by peripheral vascular resistance and endothelial function, which are directly modulated by eNOS-derived NO bioavailability. Our data demonstrate that high salt impairs eNOS activity via O-GlcNAc, leading to reduced NO-mediated vasodilation and increased peripheral resistance—effects that would be expected to predominantly elevate diastolic pressure. In contrast, systolic pressure is largely determined by cardiac output and large-artery stiffness, parameters that may be less sensitive to acute changes in endothelial function in our model. This dissociation underscores the central role of salt-induced endothelial dysfunction in altering diastolic hemodynamics and reinforces the physiological relevance of the O-GlcNAc-eNOS pathway in vascular tone regulation.

A reduction in eNOS activity along with the suppression of NO production is linked to dysfunction in endothelial cells (Chen et al., 2009). O-GlcNAc, which is a dynamic modification that occurs post-translationally, is recognized for its significant role in how cells respond to different stressors (Zachara et al., 2004). Previous studies have shown that inhibition of O-GlcNAc may augment eNOS activity. To explore the molecular mechanisms underlying high salt-induced vascular injury, we focused on the glycosylation modification of eNOS. Western blot analysis of aortic tissue and BAECs demonstrated that HSD or NaCl treatment significantly enhanced O-GlcNAc modification of eNOS and elevated OGT expression, while OGA and total eNOS levels remained unchanged (Figure 3). This suggests that eNOS activity is modulated via post-translational glycosylation, potentially leading to its uncoupling and reduced NO production. Indeed, NO levels in both serum and cell supernatant were significantly reduced following high salt exposure, confirming impaired eNOS functionality.

The most compelling finding of this study is the ability of OSMI-1, a selective OGT inhibitor, to reverse salt-induced endothelial dysfunction. In HSD-fed mice, OSMI-1 treatment significantly improved diastolic blood pressure, restored ACh-mediated vasorelaxation, and reversed vessel remodeling (Figures 4A–F). Mechanistically, these protective effects may be attributed to the suppression of OGT expression, leading to reduced eNOS O-GlcNAc levels (Figures 4G–I). These results are conceptually aligned with findings from models of diabetic atherosclerosis, where restoring eNOS coupling through folic acid supplementation or DHFR overexpression alleviates vascular damage (Beleznai and Bagi, 2012). Our data now extend this paradigm to salt-induced endothelial injury and provide the first evidence that targeting O-GlcNAc may be a viable strategy for maintaining endothelial function under dietary stress.

In the present study, we observed that neither the eNOS dimer/monomer ratio nor Caveolin-1 expression was altered under high-salt conditions, both *in vitro* and *in vivo*. Interestingly, high salt markedly reduced p-eNOS Ser1177. These findings suggest that the decrease in eNOS activity is not mediated by changes in dimerization or Caveolin-1 expression. Instead, the reduction in p-eNOS Ser1177 may be due to competitive inhibition by O-GlcNAcylation, which is consistent with previous reports indicating reciprocal regulation between O-GlcNAc modification and phosphorylation on eNOS (Aulak et al., 2020). This mechanism likely contributes to the observed decrease in NO bioavailability and endothelial dysfunction under high-salt conditions.

Several limitations of this study should be considered. First, our investigation utilized only male animals and a relatively short-term (4 weeks) intervention, leaving open questions regarding sex-specific effects and long-term adaptations. Second, while bovine aortic endothelial cells provide a valuable model system, their translational relevance to human vascular biology requires further validation. Third, although OSMI-1 has demonstrated specificity in previous studies, we cannot exclude potential off-target effects that might contribute to the observed phenotypes; future studies employing genetic approaches such as OGT knockdown would help confirm the specificity of our findings. Finally, our mechanistic analysis would be strengthened by including assessments of eNOS phosphorylation at Ser1177 (Beleznai and Bagi, 2012; Chae et al., 2025), BH4 bioavailability, and ROS production, which would provide deeper insights into the precise molecular switches between eNOS coupling and uncoupling. Beyond these limitations, it is important to consider that salt-induced endothelial dysfunction involves multiple interconnected pathways, including oxidative stress, ADMA accumulation, and activation of PARP, TRPM2, and inflammasome signaling. The O-GlcNAc pathway identified here may function in parallel to or interact with these established mechanisms, potentially serving as an integrative hub that coordinates cellular stress responses with vascular function. Future research should explore these potential intersections to develop a more comprehensive understanding of salt-induced vascular pathology.

## Conclusion

In summary, our study demonstrates that excessive salt intake leads to vascular endothelial dysfunction and diastolic blood pressure elevation through enhanced O-GlcNAc of eNOS. This modification is driven by the upregulation of OGT, resulting in reduced NO bioavailability and impaired endothelium-dependent vasodilation. Pharmacological inhibition of OGT with OSMI-1 effectively restored endothelial function both *in vivo* and *in vitro*, highlighting a novel and targetable molecular mechanism underlying salt-induced vascular injury. These findings suggest that targeting eNOS O-GlcNAc represents a promising therapeutic approach for the prevention and management of salt-sensitive hypertension.

## Data Availability

The original contributions presented in the study are included in the article/Supplementary Material, further inquiries can be directed to the corresponding author.
