# Supplementary material for: O-GlcNAcylation of eNOS in high-salt-induced thoracic aorta endothelial dysfunction in mice
Source: Front Pharmacol. 2025 Dec 16;16:1730447. doi: 10.3389/fphar.2025.1730447 (PMC12749180; doi:10.3389/fphar.2025.1730447)
Supplement: Supplementary file 1 [file DataSheet1.pdf]

Supplemental S1

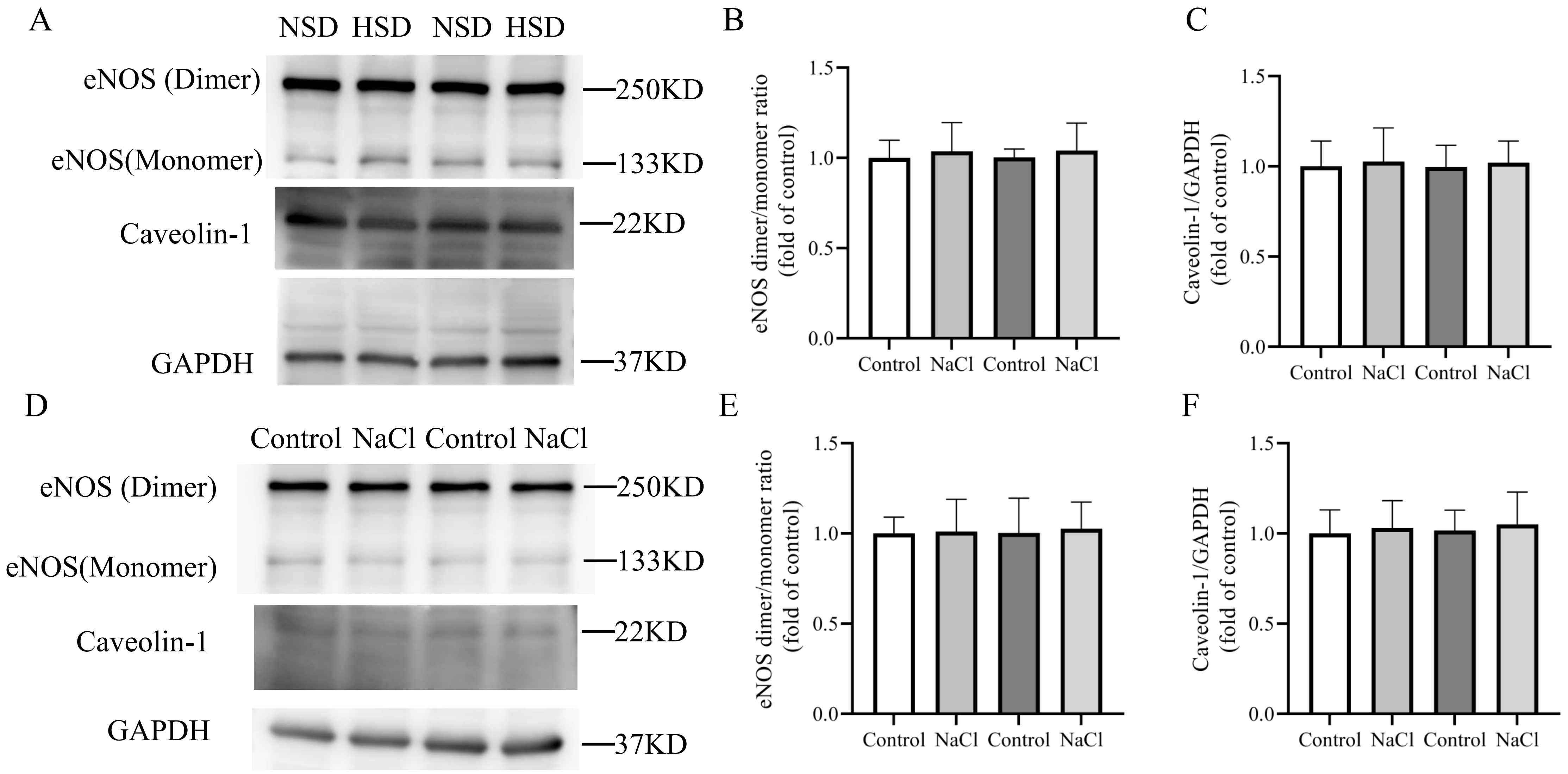

Figure S1. Assessment of eNOS dimerization and Caveolin-1 expression in vivo and in vitro. (A) Representative Western blot of eNOS dimer/monomer and Caveolin-1 protein levels in aortic tissues from mice fed NSD and HSD. (B) Quantitative analysis of the eNOS dimer/monomer ratio. (C) Quantitative analysis of Caveolin-1 protein expression normalized to GAPDH. (D) Representative Western blot of eNOS dimer/monator and Caveolin-1 in BAECs treated with normal (Control) or high-salt (NaCl) medium. (E) Quantitative analysis of the eNOS dime/monomer ratio. (F) Quantitative analysis of Caveolin-1 protein expression normalized to GAPDH. N=3. Data are mean ±SEM

Supplemental S2

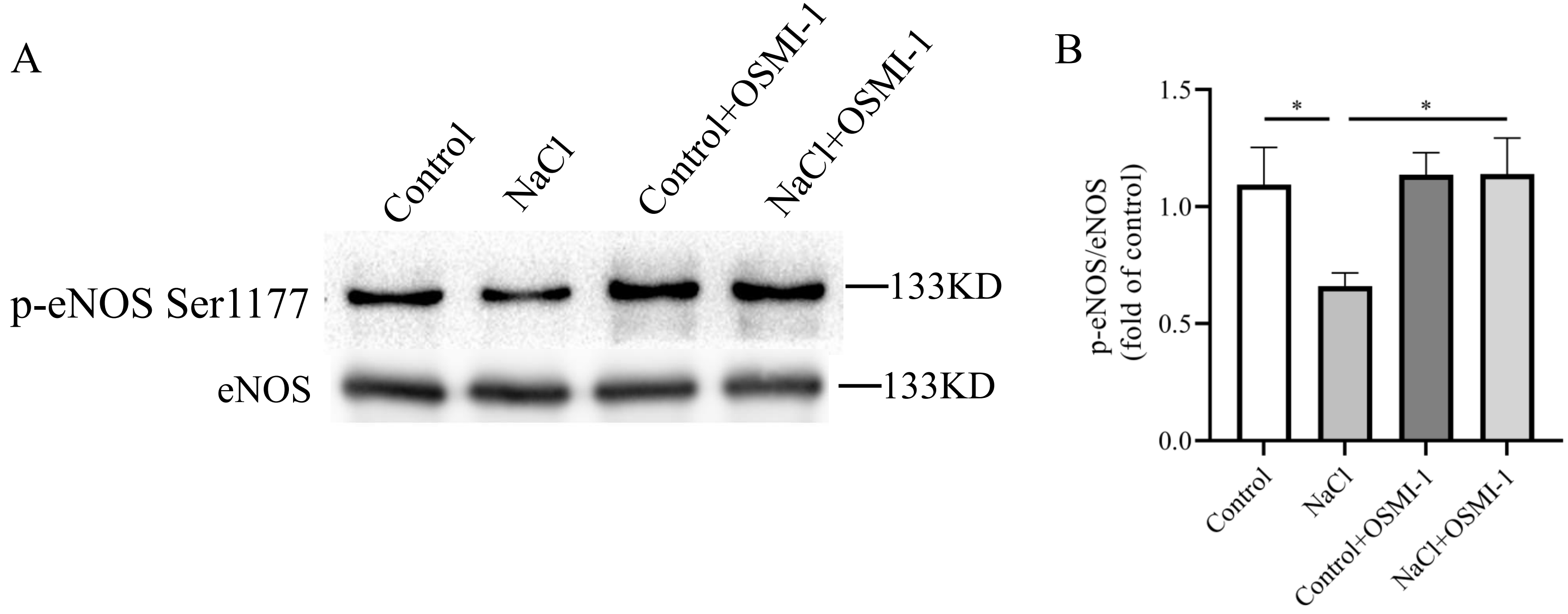

Figure S2. Effects of high-salt treatment and OGT inhibition on eNOS Ser1177 phosphorylation in BAECs. (A) Representative Western blot images of total eNOS and p-eNOS Ser1177 in BAECs under four groups: Control, NaCl, Control + OSMI-1, and NaCl + OSMI-1. (B) Quantitative analysis of p-eNOS Ser1177 /eNOS. N=3. **P*<0.01, Data are mean ±SEM

Supplemental S3

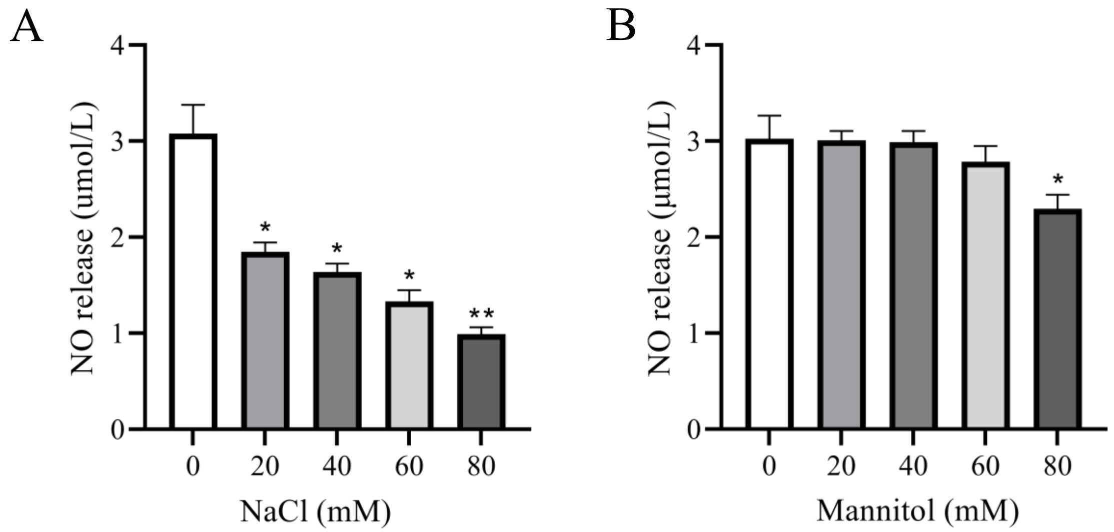

Figure S3. Detection of NO production in BAECs. (A) Measurement of NO levels in the culture supernatant of BAECs treated with 20, 40, 60, and 80 mM NaCl. (B) Measurement of NO levels in the culture supernatant of BAECs treated with 20, 40, 60 and 80 mM mannitol. N=5. *P<0.05, **P<0.01, Data are mean ±SEM.
